# Impact of the front-of-pack 5-colour nutrition label (5-CNL) on the nutritional quality of purchases: an experimental study

**DOI:** 10.1186/s12966-016-0416-4

**Published:** 2016-09-20

**Authors:** Chantal Julia, Olivier Blanchet, Caroline Méjean, Sandrine Péneau, Pauline Ducrot, Benjamin Allès, Léopold K. Fezeu, Mathilde Touvier, Emmanuelle Kesse-Guyot, Eric Singler, Serge Hercberg

**Affiliations:** 1 Université Paris 13, Equipe de Recherche en Epidémiologie Nutritionnelle (EREN), Centre d’Epidemiologie et Biostatistiques Sorbonne Paris Cité (CRESS), Inserm U1153, Inra U1125, Cnam, COMUE Sorbonne-Paris-Cité, 74 rue Marcel Cachin, Bobigny, Cedex F-93017 France; 2 Département de Santé Publique, Hôpital Avicenne (AP-HP), Bobigny, F-93017 France; 3 In Vivo BVA, Boulogne-Billancourt, France

**Keywords:** Front-of-pack nutrition label, Purchases, Nutrient profiling system, Food policies

## Abstract

**Background:**

Front-of-pack (FOP) nutrition labelling has received growing attention from public health authorities, as a tool to promote healthier diets in the population. Recently, the French Health law introduced the principle of implementing a FOP nutrition labelling system. A scientific proposal has put forward the 5-Colour Nutrition Label (5-CNL), a five-category colour-coded summary system supported by research suggesting that it is well perceived and understood in the population. Our objective was to investigate the impact of the 5-CNL on the nutritional quality of purchases in experimental supermarkets.

**Methods:**

Participants (*n* = 901) were recruited using quota sampling between September and December 2015 and evenly distributed in three experimental conditions: 1) control situation; 2) Application of the 5-CNL on all food products in three specific sections: breakfast cereals, sweet biscuits and appetizers; 3) introduction of the 5-CNL accompanied by consumer information on use and understanding of the label. Main outcome was the nutritional quality of the shopping cart in the three sections combined, measured using the United Kingdom Food Standards Agency nutrient profiling system (FSA score).

**Results:**

Significantly higher mean nutritional quality of the purchased items per section were observed for the sweet biscuits category in the intervention combining the label + communication (overall FSA score 21.01 vs. 20.23, *P* = 0.02). No significant effects were observed for the general mean over the three sections combined or other food categories. The results observed on purchase may be related to the high level of recall, self-reported and objective understanding of the label that were observed in the intervention groups as they are pre-requisites for use of a label in purchasing situations.

**Conclusion:**

These results suggest that the 5-CNL FOP nutrition label may have a limited impact on purchases, leading to healthier food choices in some food categories such as sweet biscuits.

**Trial registration:**

The trial was registered on Clinicaltrials.gov under the number NCT02546505.

**Electronic supplementary material:**

The online version of this article (doi:10.1186/s12966-016-0416-4) contains supplementary material, which is available to authorized users.

## Background

Chronic diseases represent a major burden to industrialized countries, and the epidemiologic transition in the developing world holds out the prospect of their becoming a major issue worldwide [1, 2]. Nutrition has been identified as a key modifiable lever for the prevention of chronic diseases, and national nutrition programs including multi-level and multifaceted interventions have emerged as major components of a policy aiming at reducing their burden in the population [3–5].

Front-of-pack (FOP) nutrition labelling has received growing attention from public health authorities, as a tool to promote healthier diets in the population [6, 7]. Recent reviews have highlighted the potential of FOP nutrition labelling for the promotion of healthier food choices at the point of purchase and for the improvement of the food supply towards healthier products through reformulation of existing foods and innovation [8–12]. Several countries have included a FOP nutrition label in the framework of their national nutrition programs, highlighting the interest such interventions stir in public health policies [13–16]. Recently, the French Ministry of Health has supported the principle of a FOP nutrition labelling in the 2016 Health Law, following a report from the president of the French National Health and Nutrition Program [17]. Though the law does not encompass the specific format of the label, the report from Pr. Hercberg included a proposal, the 5-Colour Nutrition Label (5-CNL), a five-category colour-coded summary system [18]. This label gives an overall appraisal of the nutritional quality of a food or beverage (except alcoholic beverages), based on the British Food Standards Agency (FSA) nutrient profiling system (FSA score) [19–21]. It takes into account in a single score the content (for 100 g) in energy (KJ), saturated fat (g), sugar (g), sodium (mg), fibers (g), proteins (g) and fruit and vegetables (%) in a given food or beverage. Feasibility of the use of the FSA score for FOP nutrition labelling was considered satisfactory and cut-offs were set for the 5 classes of the 5-CNL, with an across-the-board approach (i.e. foods from all food groups were classified using the same algorithm, albeit a few exceptions), with specific sets of cut-offs for foods and beverages [22–26]. Research conducted on this label suggests that it is both well perceived and understood in the population, which are prerequisites for a label to be actually used in purchasing situations [27, 28]. A recent study based on an online experimental supermarket found that the 5-CNL, in comparison with other existing labels and a control situation, was the label associated with the highest nutritional quality of the shopping cart, implying that it may lead to healthier food choices at the point of purchase [29]. However, the study was based on an online grocery store, and the impact of the label may differ in actual physical environments. Online shopping environments differ in the presentation of products or promotions, and the shopping experience is carried out at home, without the distractions that might occur in actual supermarkets (music, presence of other shoppers, etc.).

Our objective was to investigate the impact of the 5-CNL on the nutritional quality of purchases in an experimental supermarket environment reproducing a physical grocery shop. As use of the label is conditioned by the ability of it to draw awareness and its understanding by consumers [10], data on awareness and understanding of the 5-CNL were also estimated.

## Methods

### Population

Participants were recruited in the street through quota sampling, based on criteria of gender (male/female) and age (18-24/25-29/30-34/35-39/40-44/45-49/50-54/55-59/60-64/≥65 years old) considering the distribution of such variables in consumers of the selected food categories.

Participants were excluded if they worked in professions linked with marketing, food industry and retail, if they did not engage personally in grocery shopping for their household, or if they shopped the selected food categories for the introduction of the FOP nutrition label less than once a year.

The study was conducted between September and December 2015.

### Design

The study relied in the comparison of three independent samples, in three experimental conditions: 1) control situation with no specific FOP nutrition label on products; 2) intervention n°1: introduction of the 5-CNL as a FOP nutrition labelling on all food products with no additional information for the consumer; 3) Intervention n°2: introduction of the 5-CNL FOP nutrition label on all food products with consumer information.

### Setting

The study was conducted in LabStores®, i.e. shopper laboratory stores, which are controlled study spaces in which a store is recreated in reality. The stores are located in urban areas, close to shopping malls, ensuring a high level of passage from a varied population. Recruitment is performed throughout the day, in shop opening hours. The lab stores mimic a store environment with real shelves, products, and cash register, and present several product categories in a realistic shopping environment (light, music), except for the absence of other customers. The stores include several aisles, and are similar in size to an average supermarket store. Types and number of products proposed may vary according to the research purpose of the study. Respondents were asked to go shopping according to a shopping instruction. The purchasing instructions given (carrying out shopping for the selected categories of product, buying the products as though shopping in your usual store) were sufficiently clear to induce a category visit but broad enough not to induce a particular behaviour or brand choice. Consumers were asked to take a shopping cart and make their purchases on their own, the entire sequence being recorded by video cameras and purchases being listed as the consumers went through a fictitious checkout. At the end of the shopping session, the respondents proceeded to the cashier but didn’t actually pay for their purchases.

### Intervention

#### Products

Three frequently consumed categories of food products were selected, based on expected variability in nutritional quality of the products and their long-term conservation: breakfast cereals, sweet biscuits and appetizers (including extruded snacks, pretzels, crackers or crisps). The selected products were actual retailers’ or national brand products available on the French market. For breakfast cereals, 82 references were proposed, from 'Green'-labelled products (*N* = 3) to 'Pink'-labelled products (*N* = 29). For biscuits, 130 references were proposed, mainly labelled as 'Red' (*N* = 107). Finally, for appetizers, 84 references were proposed, from 'Orange'-labelled products (*N* = 11) to 'Red'-labelled products (*N* = 26).

#### FOP nutrition label

The 5-CNL label was proposed to be introduced to the French market to guide consumer food choices, based on a review of the literature [18]. The label is based on the Foods Standards Agency nutrient profiling system (FSA score) [20, 21], currently used in the United Kingdom by the Office of Communication for regulation of advertising to children in the UK. It provides information about the overall nutritional quality of a given food item (see Additional file 1: Figure S1). The label is represented by a scale of five colours (from green to red) with corresponding letters (from A to E) [18]. The 5-CNL label is attributed depending on the FSA score of each food: ‘Green’ (−15 to −1 points), ‘Yellow’ (0 to 2 points), ‘Orange’ (3 to 10 points), ‘Pink’ (11 to 18 points) and ‘Red’ (> = 19 points) [23] (see Additional file 1: Figure S1). The bandings for the 5-CNL allocation were determined by the High Council for Public Health, based on the distribution of the FSA score in French foods and beverages [23]. The labels were affixed using adhesive tags on the front of pack of each of the selected products. The allocation of the 5-CNL was based on nutritional data from the back of package of each food.

#### Consumer information

The third arm (termed label + communication) combined the introduction of the 5-CNL FOP nutrition label and the deliverance of consumer information, corresponding to a leaflet explaining: the introduction of the FOP nutrition label; the way the FOP nutrition label could be used to compare products; and the way the colour is attributed (see Additional file 1: Material). Information was distributed at the beginning of the shopping session, along with other information on products/brands.

### Ethics

The study protocol was approved by the Institutional Review Board of the French Institute for Health and Medical Research (n°15-248) and the Commission Nationale Informatique et Libertés (n°1821532 V0). The trial was registered on Clinicaltrials.gov under the number NCT02546505. All participants gave their written consent to participate in the study.

### Outcomes

The main outcome variable was the mean nutritional quality of the items from the selected food categories in the shopping cart, based on the FSA score. All items from the selected categories of food for the intervention were taken into account in the outcome (breakfast cereals, sweet biscuits and appetizers). Briefly, the FSA score for foods and beverages was computed taking into account nutrient content for 100 g, as mentioned in the nutritional declaration in the back of the package [21]. It allocates positive points (0–10) for content in energy (KJ), total sugar (g), saturated fatty acids (g) and sodium (mg). Negative points (0–5) are allocated to content in fruits, vegetables, legumes and nuts (%), fibers (g) and proteins (g). Scores for foods and beverages are therefore based on a discrete continuous scale from −15 (most healthy) to +40 (less healthy) (see Additional file 1: Figure S1). Therefore the lower the FSA score, the healthier the food item. The main outcome was computed as the mean of FSA scores (for 100 g) of the items in the shopping cart.

The FSA score is used for the determination of the class in the 5-CNL FOP nutrition label.

Secondary outcome variables were the nutritional content of the items from the selected food categories in the shopping cart (energy, saturated fatty acids, sugars, sodium, proteins, fibers).

Additionally, data on awareness and understanding of the FOP nutrition label were collected at the end of the shopping session through questionnaires administered by an interviewer. Awareness of the label was investigated through the recall of the FOP label (Do you recall seeing a FOP nutrition label on the shelf?). Understanding was explored through self-reported (Do you think this FOP nutrition label is easy to understand? possible answers: Very easy/rather easy/Not really easy/Not at all easy to understand) and objective understanding of the label. Objective understanding was assessed by asking participants to rate the healthiness of two products after exposure to two products presenting the label for 2 seconds (Among the two products I showed you, which do you believe is the healthier one?).

### Statistical analyses

Sample size was calculated taking into account a power of 0.80 and a type I error of 0.025 (given the three-arm design of the study) with a hypothesized effect size of 0.25 based on previous research [29]. The required sample size was 918.

The overall nutritional quality of the items in the shopping cart was assessed using the mean FSA score of the items purchased from the selected categories at the individual level, and individual means were compared across the intervention groups using ANOVAs. Two-by-two comparisons between experimental conditions were conducted using t-tests adjusted for multiple comparisons with Bonferroni corrections. Comparisons were made considering all purchased items and by product category. Comparisons by category only considered consumers of the said category of products. When significant differences were observed across intervention groups, the nutrient content of the shopping cart was compared across intervention groups. Awareness and understanding of the label across intervention groups were compared using Chi-square tests.

Weighting of the data was applied in all analyses to take into consideration the quota method of recruitment.

All tests were two-sided and a *P* value <0.05 was considered significant. Statistical analyses were performed using SAS Software (version 9.3, SAS Institute Inc, Cary, NC, USA).

## Results

Nine hundred one subjects were recruited to participate in the study, 300 in the first group, 301 in the second group and 300 in the last group. There were no significant differences in the socio-demographical characteristics of the subjects according to the intervention group (Table 1). The overall nutritional quality of the shopping cart was not significantly higher in the interventions than in the control situation (Table 2). However, significant differences in the nutritional quality of the purchased items were observed for sweet biscuits between the control situation and the intervention combining the label + communication, with a significantly higher nutritional quality observed in the intervention group (mean FSA score for sweet biscuits 21.01 vs. 20.23, *P* = 0.02). The number of articles purchased was not lower in the intervention groups (total number of purchases = 288 in the control group vs. 318 in the label + communication group). In the breakfast cereal category, mean nutritional quality was higher, but not significantly in the intervention groups. In the appetizers category, there was no significant difference in the mean nutritional quality in the intervention groups compared to the control situation (Table 2). When considering the nutrient content of purchases in the sweet biscuits category, nonsignificant lower contents in sugar and sodium were observed, as well as non-significant higher fibers content (Table 3). Pre-requisites for the use of a label in purchasing situations (i.e. awareness and understanding), may be used to interpret these results. Recall of the label was not significantly different between the control and the label only condition. However, recall was significantly higher in the label + communication group compared to the label only group (Table 4). Self-reported and objective understanding of the label were also significantly higher in the intervention groups, and more importantly in the label + communication group (Table 4).

**Table 1:** Characteristics of the sample included in the analyses according to the three experimental conditions

	Unweighted							Weighted			
	Control		Label		Label + communication			Control	Label	Label + Communication	
	*N*	%	*N*	%	*N*	%	*P*	%	%	%	*P*
Sex											
Men	73	24.33	90	29.9	99	33		30	30	30	
Women	227	75.67	211	70.1	201	67	0.06	70	70	70	1
Age, years											
18-34	133	44.33	144	47.84	127	42.33		44.01	48.37	42.59	
35-49	84	28	95	31.56	89	29.67		27.97	31.86	27.93	
> = 50	83	27.67	62	20.6	84	28	0.21	28.02	19.77	29.48	0.07
Profession											
Farmer	0	0	1	0.33	1	0.33		0	0.26	0.28	
Intermediate professions	20	6.67	23	7.64	22	7.33		6.25	7.87	7.33	
Executive	17	5.67	12	3.99	20	6.67		5.59	4.12	6.35	
Employee	105	35	117	38.87	124	41.33		34.88	39.16	40.79	
Manual worker	6	2	2	0.66	8	2.67		2.29	0.62	2.42	
Retired	36	12	32	10.63	35	11.67		11.22	9.57	12.73	
No activity	116	38.67	114	37.87	90	30	0.4	39.77	38.4	30.11	0.32
Presence of a child in the household											
Yes	76	25.33	70	23.26	89	29.67		25	25	25	
No	224	74.67	231	76.74	211	70.33	0.2	75	75	75	1

*P* value obtained with Chi ^2^ tests. Weighted analyses take into account the quota sampling recruitment method

**Table 2:** Main outcome : overall nutritional quality of the shopping cart according to the three experimental conditions

		Mean FSA score				Total number of products purchased		
	N subjects	Control	Label	Label + Communication	*P*			
Overall purchases	901	15.33 ± 3.57	15.48 ± 3.73	14.84 ± 4.24	0.10			
Sweet biscuits	565	21.01 ± 2.57*	20.5 ± 2.82	20.23 ± 2.67*	0.02	288	297	318
Appetizers	762	16.42 ± 4.32	16.67 ± 4.65	16.47 ± 4.75	0.80	433	419	425
Breakfast cereals	752	9.6 ± 4.53	9.33 ± 5.10	9.28 ± 4.49	0.71	313	310	324

*P* value obtained with ANOVA weighted according to the quota sampling method.

*P value for two-by-two comparisons by t-tests weighted according to the quota sampling method with a Bonferroni correction <0.05

**Table 3:** Energy and nutrient content of the shopping cart for the sweet biscuits category across the three experimental conditions

	Control	Label	Label + Communication	*P*
Energy (KJ/100 g)	495.5	493.3	495.2	0.5
Saturated fat (g/100 g)	12.5	12.1	12.4	0.29
Sugar (g/100 g)	33.5	33.1	32.5	0.2
Sodium (mg/100 g)	245.9	255.1	233.1	0.13
Fibres (g/100 g)	2.88	3.15	3.16	0.07
Proteins (g/100 g)	6.26	6.25	6.19	0.73

*P* value obtained with ANOVA weighted according to the quota sampling method.

**Table 4:** Recall, self-reported and objective understanding of the label according to the three experimental conditions

	Control	Label	Label + Communication	*P*
Recall of the label *(Do you recall seeing a front-of-pack label on products ?)*				
Sweet biscuits category	12.12	18.59	69.29	<0.0001
Appetizers category	18.89	16.05	65.06	<0.0001
Breakfast cereals category	19.92	25.04	55.05	<0.0001
Objective understanding *(Among the two products I showed you, which do you believe is the healthier one?)*				<0.0001
Selection of the product with the highest nutritional quality (right answer)	28.04	31.85	40.11	
Selection of the product with the lowest nutritional quality (wrong answer)	43.9	29.8	26.3	
Don’t know	41.48	38.63	19.89	
Self-reported understanding *(Do you think this FOP nutrition label is easy to understand ?)*				<0.0001
Very easy	20.42	27.22	52.36	
Rather easy	33.44	30.9	35.66	
Not really easy	42.4	35.28	22.32	
Not at all easy	38.73	40.85	20.42	

Values are percentages. *P* value obtained with Chi-Square tests. Analyses are weighted taking into account the quota sampling recruitment method

## Discussion

Our results show that an intervention combining the introduction of the 5-CNL FOP nutrition label and a communication leaflet explaining its use was associated with a higher nutritional quality of the purchases for sweet biscuits. Notably, the number of items purchased in this category was higher in the intervention group. Results were not statistically significant for the other categories of foods tested (namely, breakfast cereals and appetizers). Results from awareness and understanding variables show that the 5-CNL was highly recalled and understood in the sample population, especially in those who received the communication leaflet.

Our findings are consistent with those of a recent randomized trial testing several FOP systems currently in use in the world, including the 5-CNL [29]. The study was conducted in an experimental online supermarket where all products exhibited the label (5-CNL, other FOP nutrition labels or none for the control group). This first intervention study investigating the impact of the 5-CNL showed that it was significantly associated with a higher overall nutritional quality of the shopping cart. Overall, intervention studies on the impact of other FOP nutrition labels on purchases have shown mixed results [12, 30, 31]. To our knowledge, aside from the above-mentioned experimental study, only one short-term intervention study by Sonnenberg et al. set in a hospital cafeteria was able to show significant improvements in the nutritional quality of purchases when applying a FOP nutrition label, but only in the fraction of the population that was aware of its existence [32]. Other positive results come from experimental or observational studies with long-term evaluation of the outcome (more than a year) [33, 34]. In all other cases, the introduction of a FOP nutrition label did not significantly improve the nutritional quality of individual purchases or overall sales of healthier products [35–39].

Some hypotheses could help explain these results. In the short-term studies showing positive findings [29, 32], the interventions included the affixing of the FOP system on all products, while in other interventions, the label was affixed to 30 % of products at most [34–39]. Awareness of the label is considered an essential prerequisite for its use by consumers in purchasing situations [10]. When the label is affixed only in a portion of the food items, consumer awareness of the label is likely to be lower than when all products present the label. Moreover, the repetition of exposure to the FOP nutrition label over time is likely to increase awareness gradually. Therefore, short-term interventions are less likely to show significant positive results, all the more if only a portion of food items present a FOP nutrition label.

Finally, even in a condition where all the products from a specific shelf are labelled, the observed effect size can be considered as small: in our study, the largest observed effect size was 0.30 for biscuits. This observed effect size is consistent with the overall effect size observed in the online supermarket trial (0.25) [29]. Therefore, large sample sizes are required to find potential effects on choice. The small effect sizes observed highlight the fact that the introduction of a FOP nutrition label as a public health measure should not be considered as a ‘magic bullet’, but could only be one of many combined intervention aiming at improving the healthiness of the diet in the population, in the framework of larger public health nutrition programs [3, 5], as in itself the measure has a limited impact on purchases. However, changes of small magnitude in nutrition have shown to have long-term significant effects. For example, it has been shown that energy intakes exceeding expenditures by 5 % alone can be considered responsible of significant weight gains in the long term [40]. Moreover, the introduction of a FOP nutrition label is also suggested to entice manufacturers to reformulate existing products towards healthier compositions [41], therefore strengthening the overall effect of a label beyond its primary goal in helping food choice. The fact that the introduction of the label did not lead to a decrease in the number of items purchased, but rather to an increase should also be mentioned as a possible incentive for manufacturers to enter the scheme.

Additionally, the level of understanding can be considered as high, given that the 5-CNL is yet an experimental FOP nutrition label that has not been broadly disseminated on food packages. Moreover, objective understanding in our study was assessed after only two seconds of exposure to the label, which highlights its immediate impact, useful in purchasing situations when choice of food is made in an average 35 seconds [42]. These results are in line with other studies focusing on the comparative understanding of the 5-CNL with other FOP nutrition labelling systems, which showed the 5-CNL was the most easily understood [27, 28]. Indeed, some characteristics of the label itself have been highlighted as promoting awareness and understanding, which determine the capacity of the consumer to effectively use a label in a purchasing situation [12]. The use of colour is thought to stimulate both [11, 12, 43], in particular when using green and red cues, which are immediately used as alert signals by consumers [44]; the use of summary systems rather than nutrient-specific schemes is also likely to improve awareness and understanding [45, 46]. Noteworthy, the study by Sonnenberg et al. was also based on a colour-coded summary FOP labelling system [32].

Finally, it is of importance that significant results were only observed in the intervention combining both the introduction of the label and a communication leaflet prompting consumer attention on the existence of a label and its use in purchasing situations. The abundance of information that is found in a shopping environment often impairs the capacity of the consumer to notice a nutrition label, in particular in time constraining conditions [47]. Accompanying the introduction of the label with a communication leaflet therefore increases the awareness of the consumer, and probably acts to prompt his active seeking of the information to use it [10]. Such results tend to emphasize the importance of wide multimedia communication campaigns to accompany the introduction of a FOP label nationally, to ensure high awareness in the consumer, which precedes active use of the label in the population.

Our results also show that the effect of the 5-CNL label was differential according to the type of product considered. These results are in line with those of Waterlander et al., which showed some differences of the interventions according to the food category considered, though overall impact of the label was not significant [39]. Given the variability in the nature and quality of the foods and beverages offer, the reception and use of a FOP nutrition labelling system is also likely to vary across food categories. Use of a FOP nutrition label has been repeatedly associated with health concerns in the consumer [12, 34]. Moreover, value placed on prior preconceptions about the nutritional quality of foods may have a different importance depending on the food category considered [48]. Therefore, health perceptions and expectations towards a food category could also impact the likelihood of using the label in purchasing situation. This could explain in part the absence of an effect in the appetizers category. It may be inferred that prior preconceptions of consumers place appetizers in a ‘less healthy’ category, and that health expectations in this category are low. Therefore, FOP nutrition labelling in this category may have been overlooked. The absence of any significant impact on the breakfast cereals category in this instance is less easily explained. Breakfast cereals display a large variety in nutritional quality, which is made apparent by the label [26]. Moreover, they are perceived as healthy components of a diet [49]. The impact of the label in this category is therefore much lower than expected. This may be in part due to the variability of the FSA score in the category, which yields a large standard deviation (SD = 4.62 in breakfast cereals vs. 2.74 in sweet biscuits), and therefore a lower power to detect a significant difference in this case.

Our study is subject to some limitations. First, the recruitment method used a quota sampling which ensured the inclusion of consumers of all the categories of products selected for the intervention. Moreover, we showed that the impact of the label was differential across categories of foods. Generalization of results to other foods or beverages and to other profiles of consumers needs to be addressed cautiously. However, results are consistent with those of the study investigating the impact of the 5-CNL in an experimental online supermarket, which included several other categories of foods [29]. Secondly, our study was carried out in experimental conditions, in LabStores®. Though the LabStores® are designed to reproduce actual shopping conditions, the absence of usual disturbances present in shops (e.g. the absence of other shoppers or promotions) may have influenced the behavior of the participants. Moreover, the number of categories proposed is lower than a typical supermarket. It has been shown that the number of products proposed can impact the healthfulness of the choice at the point of purchase, more importantly than the presence of a FOP nutrition label [35]. However, in our case, the number of products proposed to the consumer was similar to choice afforded by a supermarket, with more than 80 references in each category. Last, though they were not aware that they would not have to pay for the products at the end of the experiment, subjects were aware of participating in a marketing research study, which might amplify the attention they place in the information provided in the environment. Participants in the study may therefore have been more aware of the presence of the label, and therefore more likely to use it. However, effect sizes observed in our study are comparable to other studies [29], limiting the importance of this bias. Generalization of our results should however be made with caution, given these limitations to the study.

## Conclusion

Our study suggests that the 5-CNL FOP nutrition label may lead to healthier food purchases in some food categories such as sweet biscuits. The quick understanding of the label may act as a driver for this effect. However, the effect sizes observed are small, which tend to suggest that this type of measure can only be one of many in a multifaceted nutrition program.

## Additional file

Additional file 1: Figure S1. (FSA score computation and 5-CNL attribution) and Supplemental material (communication leaflet). (ZIP 464 kb)

## Abbreviations

5-CNL: Five-colour nutrition label
DI: Dietary index
FSA: Food standards agency
FSA-NPS DI: Food standards agency nutrient profiling system dietary index
NPS: Nutrient profiling system
PNNS: Programme National Nutrition Santé

## References

[1] World Health Organization (2003). Diet, Nutrition and the prevention of chronic diseases. Report of a joint WHO/FAO expert consultation.

[2] World Health Organization (2009). Global Health Risks.

[3] Hughes R (2004). Competencies for effective public health nutrition practice: a developing consensus. Public Health Nutr.

[4] Lachat C, Van Camp J, De Henauw S (2005). A concise overview of national nutrition action plans in the European Union Member States. Public Health Nutr.

[5] Serra-Majem L (2009). Moving forward in public health nutrition - the I World Congress of Public Health Nutrition - Introduction. Nutr Rev.

[6] Organization for Economic Co-operation and Development. Promoting sustainable consumption – good practices in OECD countries. 2008. Paris.

[7] World Health Organization. Global strategy on diet, physical activity and health. 2004. Geneva.

[8] Campos S, Doxey J, Hammond D (2011). Nutrition labels on pre-packaged foods: a systematic review. Public Health Nutr.

[9] Cowburn G, Stockley L (2005). Consumer understanding and use of nutrition labelling: a systematic review. Public Health Nutr.

[10] Grunert KG, Wills JM (2007). A review of European research on consumer response to nutrition information on food labels. J Public Health.

[11] Hawley KL, Roberto CA, Bragg MA, Liu PJ, Schwartz MB, Brownell KD (2013). The science on front-of-package food labels. Public Health Nutr.

[12] Hersey JC, Wohlgenant KC, Arsenault JE, Kosa KM, Muth MK (2013). Effects of front-of-package and shelf nutrition labeling systems on consumers. Nutr Rev.

[13] Asp NG, Bryngelsson S. Health claims in the labelling and marketing of food products: the Swedish food sector’s Code of Practice in a European perspective. Food &amp; Nutrition Research; Vol 51, No 3 (2007) 2007.

[14] Choices International Foundation. Product Criteria v2.2. 2011. 18-6-2014.

[15] Commonwealth of Australia. Health Star Rating System. A joint Australian, state and territory governments initiatives in partnership with industry, public health and consumer groups. 2015. Commonwealth of Australia. 22-4-2015.

[16] Food Standards Agency. Signposting and traffic light labeling. 2010. London, FSA. 10-2-2015.

[17] Touraine, Marisol. Projet de loi n°2302 relatif à la Santé, présenté au nom de M. Manuel Valls, Premier ministre, par Mme Marisol Touraine, ministre des affaires sociales, de la santé et des droits des femmes. Exposé des motifs. 2015. Paris, Assemblée Nationale. 22-4-2015.

[18] Hercberg, S. Propositions pour un nouvel élan de la politique nutritionnelle française de santé publique dans le cadre de la stratégie nationale de santé. 1ère partie : mesures concernant la prévention nutritionnelle. 2013. Paris. 28-5-2014.

[19] Arambepola C, Scarborough P, Rayner M (2008). Validating a nutrient profile model. Public Health Nutr.

[20] Rayner, M., Scarborough, P., Stockley, L., and Boxer, A. Nutrient profiles: development of Final model. Final Report [online]. 2005. London, FSA. 16-1-2014.

[21] Rayner, M., Scarborough, P., and Lobstein, T. The UK Ofcom Nutrient Profiling Model - Defining ‘healthy’ and ‘unhealthy’ food and drinks for TV advertising to children. 2009. London, OfCom. 16-1-2014.

[22] ANSES. Evaluation de la faisabilité du calcul d’un score nutritionnel tel qu’élaboré par Rayner et al. Rapport d’appui scientifique et technique. 2015. Paris, ANSES. 9-11-2015.

[23] Haut Conseil de la Santé Publique. Avis relatif à l’information sur la qualité nutritionnelle des produits alimentaires. 2015. Paris, HCSP. 9-11-2015.

[24] Julia C, Kesse-Guyot E, Touvier M, Mejean C, Fezeu L, Hercberg S (2014). Application of the British Food Standards Agency nutrient profiling system in a French food composition database. Br J Nutr.

[25] Julia C, Ducrot P, Peneau S (2015). Discriminating nutritional quality of foods using the 5-Color nutrition label in the French food market: consistency with nutritional recommendations. Nutr J.

[26] Julia C, Kesse-Guyot E, Ducrot P (2015). Performance of a five category front-of-pack labelling system - the 5-colour nutrition label - to differentiate nutritional quality of breakfast cereals in France. BMC Public Health.

[27] Ducrot P, Mejean C, Julia C (2015). Objective understanding of front-of-package nutrition labels among nutritionally at-risk individuals. Nutrients.

[28] Ducrot P, Mejean C, Julia C (2015). Effectiveness of front-of-pack nutrition labels in French adults: results from the NutriNet-sante cohort study. Plos One.

[29] Ducrot P, Julia C, Méjean C (2016). Impact of different front-of-pack nutrition labels on consumer purchasing intentions: a randomized controlled trial. Am J Prev Med.

[30] Van ’t RJ (2013). Sales effects of product health information at points of purchase: a systematic review. Public Health Nutr.

[31] Volkova E, Ni MC (2015). The influence of nutrition labeling and point-of-purchase information on food behaviours. Curr Obes Rep.

[32] Sonnenberg L, Gelsomin E, Levy DE, Riis J, Barraclough S, Thorndike AN (2013). A traffic light food labeling intervention increases consumer awareness of health and healthy choices at the point-of-purchase. Prev Med.

[33] Sutherland LA, Kaley LA, Fischer L (2010). Guiding stars: the effect of a nutrition navigation program on consumer purchases at the supermarket. Am J Clin Nutr.

[34] Vyth EL, Steenhuis IH, Vlot JA (2010). Actual use of a front-of-pack nutrition logo in the supermarket: consumers’ motives in food choice. Public Health Nutr.

[35] Aschemann-Witzel J, Grunert KG, van Trijp HCM (2013). Effects of nutrition label format and product assortment on the healthfulness of food choice. Appetite.

[36] Freedman MR, Connors R (2010). Point-of-purchase nutrition information influences food-purchasing behaviors of college students: a pilot study. J Am Diet Assoc.

[37] Rahkovsky I, Lin B-H, Jordan Lin C-T, Lee J-Y (2013). Effects of the guiding stars program on purchases of ready-to-eat cereals with different nutritional attributes. Food Policy.

[38] Sacks G, Rayner M, Swinburn B (2009). Impact of front-of-pack ‘traffic-light’ nutrition labelling on consumer food purchases in the UK. Health Promot Int.

[39] Waterlander WE, Steenhuis IH, de Boer MR, Schuit AJ, Seidell JC (2013). Effects of different discount levels on healthy products coupled with a healthy choice label, special offer label or both: results from a web-based supermarket experiment. Int J Behav Nutr Phys Act.

[40] Brown WJ, Williams L, Ford JH, Ball K, Dobson AJ (2005). Identifying the energy gap: magnitude and determinants of 5-year weight gain in midage women. Obes Res.

[41] Vyth EL, Steenhuis IHM, Roodenburg AJC, Brug J, Seidell JC (2010). Front-of-pack nutrition label stimulates healthier product development: a quantitative analysis. Int J Behav Nutr Phys Act..

[42] Grunert KG, Fernandez-Celemin L, Wills JM, Storcksdieck Genannt BS, Nureeva L (2010). Use and understanding of nutrition information on food labels in six European countries. Z Gesundh Wiss.

[43] van Herpen E, Trijp HC (2011). Front-of-pack nutrition labels. Their effect on attention and choices when consumers have varying goals and time constraints. Appetite.

[44] Liu PJ, Wisdom J, Roberto CA, Liu LJ, Ubel PA (2014). Using behavioral economics to design more effective food policies to address obesity. Appl Econ Perspect P.

[45] Bialkova S, van Trijp H (2010). What determines consumer attention to nutrition labels?. Food Qual Prefer.

[46] Feunekes GI, Gortemaker IA, Willems AA, Lion R, Van den Kommer M (2008). Front-of-pack nutrition labelling: testing effectiveness of different nutrition labelling formats front-of-pack in four European countries. Appetite.

[47] Van Kleef E, Dagevos H (2015). The growing role of front-of-pack nutrition profile labeling: a consumer perspective on Key issues and controversies. Crit Rev Food Sci Nutr.

[48] Szanyi JM (2010). Brain food: bringing psychological insights to bear on modern nutrition labeling efforts. Food Drug Law J.

[49] Schwartz MB, Vartanian LR, Wharton CM, Brownell KD (2008). Examining the nutritional quality of breakfast cereals marketed to children. J Am Diet Assoc.

