# Supplementary material for: Impact of the front-of-pack 5-colour nutrition label (5-CNL) on the nutritional quality of purchases: an experimental study
Source: Int J Behav Nutr Phys Act. 2016 Sep 20;13:101. doi: 10.1186/s12966-016-0416-4 (PMC5028942; doi:10.1186/s12966-016-0416-4)
Supplement: Additional file 1: Figure S1. — (FSA score computation and 5-CNL attribution) and Supplemental material (communication leaflet). (ZIP 464 kb) [file 12966_2016_416_MOESM1_ESM.zip › Additional file 1/Score computation-HCSP.pptx]

## Slide 1
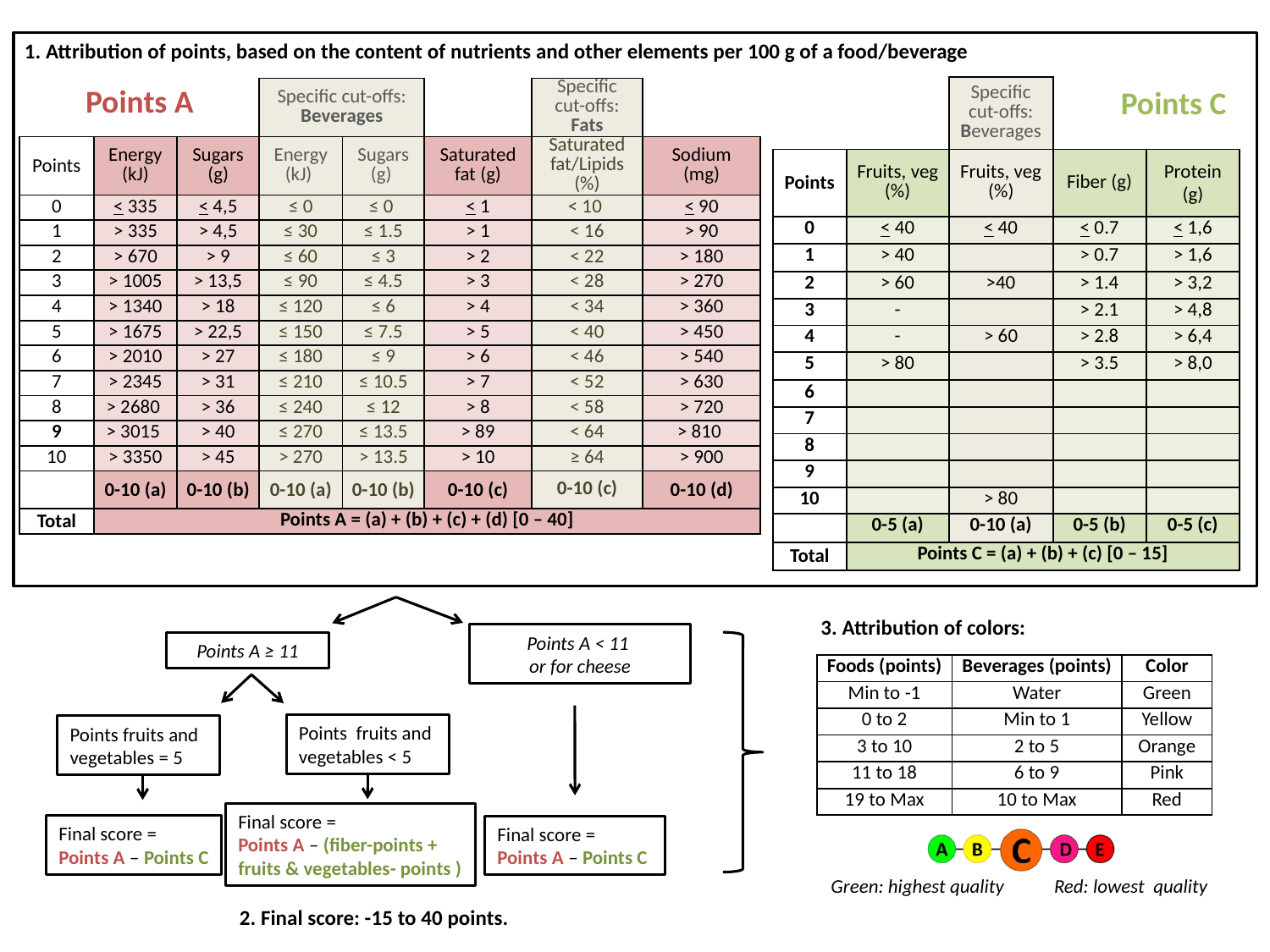

1. Attribution of points, based on the content of nutrients and other elements per 100 g of a food/beverage
Points A
| | | Specific cut-offs: Beverages | | |
| --- | --- | --- | --- | --- |
| Points | Fruits, veg (%) | Fruits, veg (%) | Fiber (g) | Protein (g) |
| 0 | < 40 | < 40 | < 0.7 | < 1,6 |
| 1 | > 40 | | > 0.7 | > 1,6 |
| 2 | > 60 | >40 | > 1.4 | > 3,2 |
| 3 | - | | > 2.1 | > 4,8 |
| 4 | - | > 60 | > 2.8 | > 6,4 |
| 5 | > 80 | | > 3.5 | > 8,0 |
| 6 | | | | |
| 7 | | | | |
| 8 | | | | |
| 9 | | | | |
| 10 | | > 80 | | |
| | 0-5 (a) | 0-10 (a) | 0-5 (b) | 0-5 (c) |
| Total | Points C = (a) + (b) + (c) [0 – 15] | | | |
Points C
| | | | Specific cut-offs: Beverages | | | Specific cut-offs: Fats | |
| --- | --- | --- | --- | --- | --- | --- | --- |
| Points | Energy (kJ) | Sugars (g) | Energy (kJ) | Sugars (g) | Saturated fat (g) | Saturated fat/Lipids (%) | Sodium (mg) |
| 0 | < 335 | < 4,5 | ≤ 0 | ≤ 0 | < 1 | < 10 | < 90 |
| 1 | > 335 | > 4,5 | ≤ 30 | ≤ 1.5 | > 1 | < 16 | > 90 |
| 2 | > 670 | > 9 | ≤ 60 | ≤ 3 | > 2 | < 22 | > 180 |
| 3 | > 1005 | > 13,5 | ≤ 90 | ≤ 4.5 | > 3 | < 28 | > 270 |
| 4 | > 1340 | > 18 | ≤ 120 | ≤ 6 | > 4 | < 34 | > 360 |
| 5 | > 1675 | > 22,5 | ≤ 150 | ≤ 7.5 | > 5 | < 40 | > 450 |
| 6 | > 2010 | > 27 | ≤ 180 | ≤ 9 | > 6 | < 46 | > 540 |
| 7 | > 2345 | > 31 | ≤ 210 | ≤ 10.5 | > 7 | < 52 | > 630 |
| 8 | > 2680 | > 36 | ≤ 240 | ≤ 12 | > 8 | < 58 | > 720 |
| 9 | > 3015 | > 40 | ≤ 270 | ≤ 13.5 | > 89 | < 64 | > 810 |
| 10 | > 3350 | > 45 | > 270 | > 13.5 | > 10 | ≥ 64 | > 900 |
| | 0-10 (a) | 0-10 (b) | 0-10 (a) | 0-10 (b) | 0-10 (c) | 0-10 (c) | 0-10 (d) |
| Total | Points A = (a) + (b) + (c) + (d) [0 – 40] | | | | | | |
Points A < 11
or for cheese
Points A ≥ 11
Points fruits and vegetables < 5
Points fruits and vegetables = 5
Final score =
Points A – Points C
Final score =
Points A – Points C
Final score =
Points A – (fiber-points +
fruits & vegetables- points )
3. Attribution of colors:
| Foods (points) | Beverages (points) | Color |
| --- | --- | --- |
| Min to -1 | Water | Green |
| 0 to 2 | Min to 1 | Yellow |
| 3 to 10 | 2 to 5 | Orange |
| 11 to 18 | 6 to 9 | Pink |
| 19 to Max | 10 to Max | Red |
Red: lowest quality
Green: highest quality
2. Final score: -15 to 40 points.
