# Supplementary figures and images for: Impact of the front-of-pack 5-colour nutrition label (5-CNL) on the nutritional quality of purchases: an experimental study
Source: Int J Behav Nutr Phys Act. 2016 Sep 20;13:101. doi: 10.1186/s12966-016-0416-4 (PMC5028942; doi:10.1186/s12966-016-0416-4)

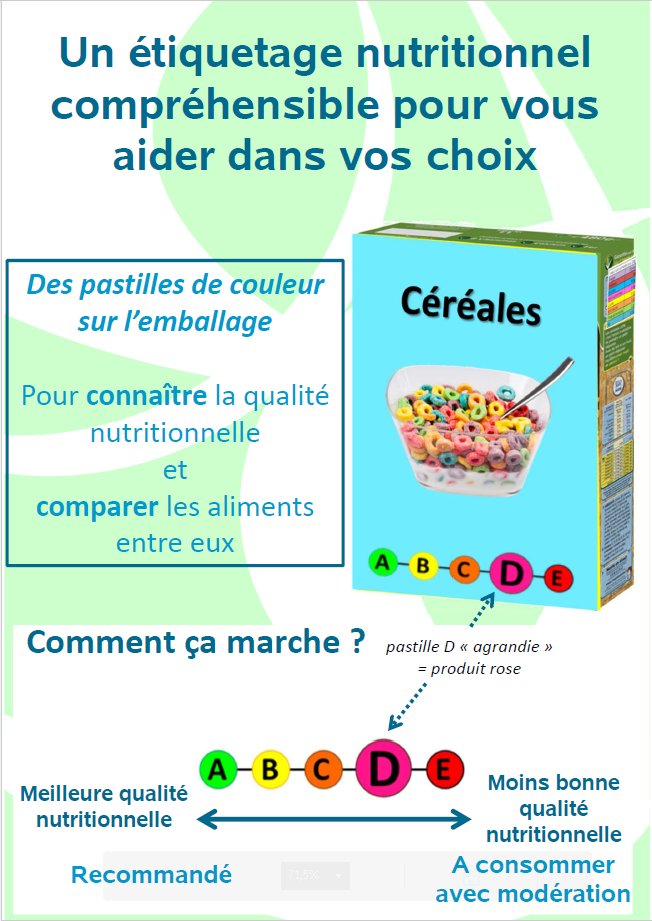

Supplement: Additional file 1: Figure S1. — (FSA score computation and 5-CNL attribution) and Supplemental material (communication leaflet). (ZIP 464 kb) [file 12966_2016_416_MOESM1_ESM.zip › Additional file 1/flyer_image avant.PNG]

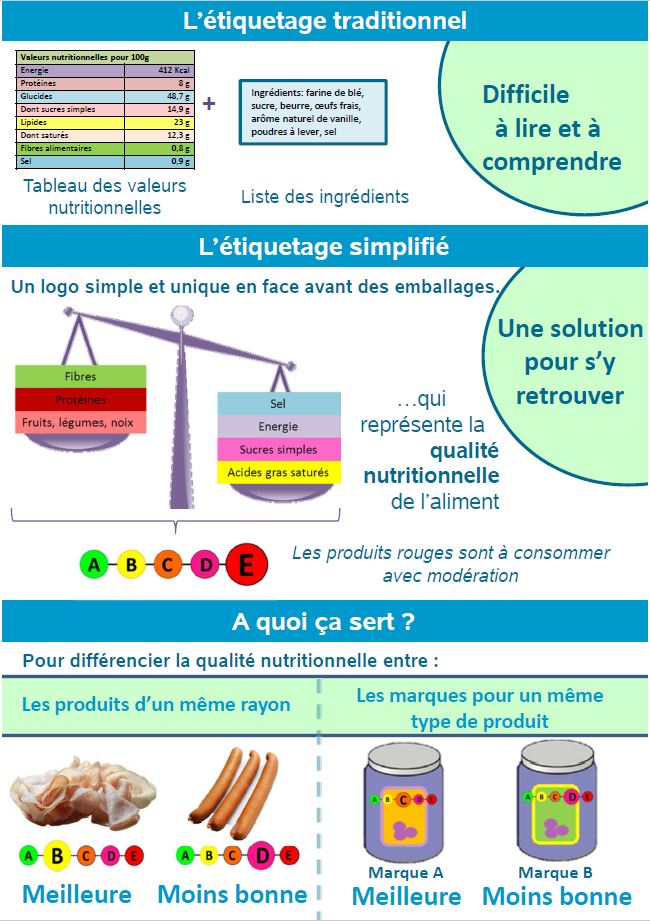

Supplement: Additional file 1: Figure S1. — (FSA score computation and 5-CNL attribution) and Supplemental material (communication leaflet). (ZIP 464 kb) [file 12966_2016_416_MOESM1_ESM.zip › Additional file 1/renamed_1e1fc.JPG]
